# Genetic characterization of a multidrug-resistant *Salmonella enterica* serovar Agona isolated from a dietary supplement in Germany

**DOI:** 10.3389/fmicb.2023.1284929

**Published:** 2023-11-15

**Authors:** Lee Julia Bartsch, Maria Borowiak, Carlus Deneke, Josephine Gruetzke, Jens-Andre Hammerl, Burkhard Malorny, Istvan Szabo, Thomas Alter, Kim Katherine Nguyen, Jennie Fischer

**Affiliations:** ^1^Department Biological Safety, German Federal Institute for Risk Assessment, Berlin, Germany; ^2^Institute of Food Safety and Food Hygiene, School of Veterinary Medicine, Freie Universität Berlin, Berlin, Germany; ^3^Bavarian Health and Food Safety Authority, Erlangen, Germany

**Keywords:** *Salmonella* Agona, dietary supplement, whole genome sequencing, antimicrobial resistance, plasmid

## Abstract

*Salmonella enterica* subsp. *enterica* serovar Agona has a history of causing food-borne outbreaks and any emergence of multidrug-resistant (MDR) isolates in novel food products is of concern. Particularly, in food products frequently consumed without sufficient heating prior to consumption. Here, we report about the MDR isolate, 18-SA00377, which had been isolated from a dietary supplement in Germany in 2018 and submitted to the German National Reference Laboratory for *Salmonella*. WGS-based comparative genetic analyses were conducted to find a potential reservoir of the isolate itself or mobile genetic elements associated with MDR. As a phylogenetic analysis did not yield any closely related *S.* Agona isolates, either globally or from Germany, a detailed analysis of the largest plasmid (295,499 bp) was performed as it is the main carrier of resistances. A combined approach of long-read and short-read sequencing enabled the assembly of the isolate’s chromosome and its four plasmids. Their characterization revealed the presence of 23 different antibiotic resistance genes (ARGs), conferring resistance to 12 different antibiotic drug classes, as well as genes conferring resistance to six different heavy metals. The largest plasmid, pSE18-SA00377-1, belongs to the IncHI2 plasmid family and carries 16 ARGs, that are organized as two distinct clusters, with each ARG associated with putative composite transposons. Through a two-pronged approach, highly similar plasmids to pSE18-SA00377-1 were identified in the NCBI database and a search for *Salmonella* isolates with a highly similar ARG resistance profile was conducted. Mapping and structural comparisons between pSE18-SA00377-1 and these plasmids and *Salmonella* isolates showed that both the plasmid backbone and identical or similar ARG clusters can be found not only in *Salmonella* isolates, originating mostly from a wide variety of livestock, but also in a diverse range of bacterial genera of varying geographical origins and isolation sources. Thus, it can be speculated that the host range of pSE18-SA00377-1 is not restricted to *Salmonella* and its spread already occurred in different bacterial populations. Overall, this hints at a complex history for pSE18-SA00377-1 and highlights the importance of surveilling multidrug-resistant *S. enterica* isolates, especially in novel food items that are not yet heavily regulated.

## 1. Introduction

The bacterial genus *Salmonella* spans two species and more than 2,500 serovars (Grimont and Weill, 2007). Non-typhoidal serovars of one of its constituent subspecies, *Salmonella enterica* subsp. *enterica*, are the principal cause of food-borne illness worldwide manifesting as enteric infections (salmonellosis) with symptoms such as diarrhea, fever, abdominal pain and vomiting. Salmonellosis is a worldwide public health issue. In 2021, it was the second most reported zoonosis with over 60,000 reported cases in humans in the European Union (EU), including 71 deaths (European Food Safety Authority and European Centre for Disease Prevention and Control, 2022b). Additionally, the World Health Organization (WHO) estimates that salmonellosis accounts for more than 150,000 deaths worldwide annually (Ao et al., 2015). Despite the large number of serovars, fewer than 100 serovars are the main causative agents of human salmonellosis.

Starting in the 1970s, the serovar *Salmonella enterica* subsp. *enterica* serovar (*S*.) Agona has emerged on the radar of public health agencies, when a major *S.* Agona outbreak in five different countries was traced to animal feed based on contaminated fish meal from Peru (Clark et al., 1973). *S.* Agona now ranks among the top 20 most frequent serovars both in the European Economic Area (EEA) and European Union, having caused 784 reported cases of human salmonellosis from 2019 to 2021 (European Food Safety Authority and European Centre for Disease Prevention and Control, 2022a). In the past decades, numerous *S.* Agona outbreaks have occurred, ranging from smaller, localized outbreaks to larger, transnational and even transcontinental outbreaks. The first recognized transcontinental *S.* Agona outbreak occurred in 1994/95 in England, Wales, the United States (Killalea et al., 1996), and Israel. Here, the origin was traced to contaminated kosher savory snacks, frequently consumed by children (Shohat et al., 1996). A further outbreak in the United States occurred in a similar isolation source, dry rice and wheat cereal products, in 1998 and re-occurred 10 years later in 2008 (Russo et al., 2013). A similar re-emergence of *S.* Agona contamination in the same food matrix and linked to the same production facility, but 12 years apart, has also been stipulated with the 2005 (Brouard et al., 2007) and 2017 (Jourdan-da Silva et al., 2018) outbreaks in infant milk formula. Other past *S.* Agona outbreaks could be traced to air-dried beef products (Taylor et al., 1998), pre-cooked meat products (Nicolay et al., 2011), fresh papaya (Mba-Jonas et al., 2018) and aniseed tea (Koch et al., 2005). Notably, these outbreaks occurred in a diverse range of mostly dried food products that are frequently consumed by children and infants without sufficient prior cooking.

Since salmonellosis symptoms are ordinarily mild, treatments usually do not include antibiotics, except for serious infections or in infections of vulnerable populations such as infants, the elderly and immunocompromised. However, excessive and improper use of antibiotics in the treatment of food-producing animals has led to the emergence of antimicrobial resistance (AMR) as well as multidrug-resistance (MDR) in *Salmonella* species. Particularly alarming is the occurrence of isolates with increased resistance to ciprofloxacin or combined resistance to fluoroquinolones and third generation cephalosporins (European Food Safety Authority and European Centre for Disease Prevention and Control, 2023). This trend poses a major threat to public health through contamination in the food chain. *Salmonella* has been shown to harbor antimicrobial resistances genes (ARGs) including those, causing resistance against last-resort antibiotics such as colistin (Borowiak et al., 2017; Fernández et al., 2018) encoded on various plasmid families (Carattoli, 2003; Rozwandowicz et al., 2018). Therefore, monitoring and surveillance of antimicrobial resistance in *Salmonella* is crucial to prevent further spread and infection. Particular attention has to be given to novel foods such as insect-derived foods or dietary supplements as they are often produced using new methods, with novel ingredients from unfamiliar sources. Especially, the recent increase in the consumption of dietary supplements in the EU (Lordan, 2021) is worrying as similar matrices have caused *Salmonella* outbreaks before – including *S.* Agona outbreaks. None of these matrices – dietary supplements, cereals, etc. – are heated or are sufficiently heated immediately prior to consumption. Thus, potential contaminations with *Salmonella* spp. pose a serious health risk to the consumer, especially since infants and vulnerable or ill populations frequently consume these products. This is evident in the 2021 outbreak of salmonellosis by *S.* Typhimurium in Denmark, which was linked to contaminated herbal supplement capsules containing psyllium husk (Technical University of Denmark, 2022).

Here, we have investigated a multidrug-resistant *S.* Agona, isolated from dietary supplements in 2018. We also sequenced and characterized its very large constituent plasmid harboring many antimicrobial and heavy metal resistance genes on mobile genetic elements and tried to reconstruct its origin. Structural comparisons revealed a composite plasmid structure, found in isolates from numerous other genera, geographic origins and isolation matrices highlighting the enormous potential for shuffling resistance determinants within an epidemiologically widely disseminated plasmid backbone.

## 2. Materials and methods

### 2.1. Strain collection and isolation

On average, the National Reference Laboratory (NRL) for *Salmonella* in Germany receives around 3,000–4,000 *Salmonella* isolates annually from different sources. Since 2018, whole-genome short-read sequencing is completed on all *S.* Enteritidis isolates, as well as isolates from national zoonosis monitoring and control programs. Since 2019, these isolates are further supplemented with sequencing of project- and outbreak-specific isolates, rough *Salmonella* isolates, and since 2020 isolates arising from Phase I of GenoSalmSurv (Uelze et al., 2021). Since 2021, foodborne isolates, isolates from Phase II of GenoSalmSurv and isolates arising from antimicrobial resistance monitoring according to Commission Implementing Decision (EU) 2020/1729 are also sequenced.

Isolate 18-SA00377 was submitted to the NRL for *Salmonella* in 2018. The sample was isolated from a dietary supplement that had been submitted to the Bavarian Health and Food Safety Authority in 2018, after having been recalled by the distributor in late 2017. According to submission metadata supplied by the Bavarian authority, the dietary supplement consisted of gelatin capsules filled with plant material enriched with vitamins and magnesium, intended for twice daily consumption.

### 2.2. Serotyping of isolates

The *S.* Agona isolates of this study underwent classical serotyping according to the White-Kauffmann-Le Minor scheme (Grimont and Weill, 2007) by slide agglutination with the O- and H-antigen specific sera (Sifin Diagnostics, Berlin, Germany) as part of the routine diagnostics of the NRL for *Salmonella*.

### 2.3. Antibiotic susceptibility testing

Antibiotic susceptibility testing was performed by the NRL for Antibiotic Resistance at the BfR, using broth microdilutions or disk diffusion. Following CLSI guidelines (version A7-M11) for broth microdilution procedures, minimum inhibitory concentrations (MIC) were obtained for 37 different antibiotics. These were interpreted following the epidemiological cutoff values provided by EUCAST (v.10.0) (The European Committee on Antimicrobial Susceptibility Testing, 2021).

### 2.4. S1-pulsed-field gel electrophoresis (S1-PFGE)

Preparation of agarose plug molds and subsequent digestion by S1 nuclease (Thermo Fisher Scientific) were performed using a previously described protocol (Rodríguez et al., 2009). A plasmid pattern by PFGE was generated using the CHEF-DR III system (Bio-Rad Laboratories, Madrid, Spain) under standardized run conditions described by PulseNet standardized protocol (available at^1^). The *Salmonella* Braenderup strain H9812 (digested with the restriction endonuclease “XbaI” (Thermo Fisher Scientific, Darmstadt, Germany)) was used for size comparisons.

### 2.5. Whole genome sequencing (WGS) of *Salmonella* Agona isolate 18-SA00377

For sequencing of 18-SA00377, genomic DNA was extracted using the PureLink Genomic DNA Mini Kit (Thermo Fisher Scientific, Waltham, MA, USA) and sequenced using MiSeq (Illumina, San Diego, CA, USA) and Oxford Nanopore Technologies (ONT, Oxford, UK) MinION devices.

A short-read sequencing library was prepared using the sparQ DNA Frag & Library Prep Kit (Quantabio Beverly, MA, USA). Paired-end sequencing was performed in 2 × 151 bp cycles on an Illumina MiSeq instrument using the MiSeq Reagent Kit v3 (600 cycle). Trimming of short-reads using fastp v0.19.5 (Chen et al., 2018) resulted in 1.9 million high quality reads (≥ 89% Q30).

An Oxford Nanopore (ONT) sequencing library was prepared according to manufacturer’s protocol using the Rapid Barcoding Kit (SQK-RBK004) and sequenced on an ONT MinION sequencer MK1C using a FLO-MIN106 R9.4.1 flow cell. Basecalling was performed with Albacore v2.3.1 (available at^2^). Obtained reads were trimmed using Porechop v0.2.3 (available at^3^), filtered, and quality checked using NanoStat v1.4.0 and NanoFilt v2.7.1 (De Coster et al., 2018), respectively. In total, 123,777 reads with a read length N50 value of 11,288 bp and a mean read quality score of 10.3 were available.

Both data sets were assembled and circularized using Unicycler v0.4.8 (Wick et al., 2017) including Pilon (Walker et al., 2014). Default parameters were used for all software unless otherwise noted.

### 2.6. Genotypic characterization and visualization

Characterization of the *S.* Agona sequences from the NRL for *Salmonella*, including 18-SA00377 and its four plasmids, was completed using the BakCharak pipeline version 3.0.4 (including -species *Salmonella* and -bakta options) (available at^4^). In short, the pipeline relies on the following tools: NCBI AMRFinderPlus (Feldgarden et al., 2021) to find antimicrobial resistance genes, ABRicate (available at^5^) to classify plasmids using CGE PlasmidFinder (Carattoli et al., 2014) and to find virulence factors using VFDB (Chen et al., 2015), Mash (Ondov et al., 2016) to find a nearest reference and plasmid reference using NCBI RefSeq (O'Leary et al., 2015) and NCBI plasmid database, respectively, Blastn (Camacho et al., 2009) to blast plasmids against the NCBI plasmid database, Bakta (Schwengers et al., 2021) for annotations using the Bakta database (Schwengers, 2021), and lastly, Platon (Schwengers et al., 2020) to predict plasmid contigs utilizing the Platon database (Schwengers, 2020). Further, *Salmonella*-specific characterization occurred using the SISTR tool with the sistr database and sistr 330 locus scheme for serotyping and cgMLST, respectively (Yoshida et al., 2016).

The annotations were supplemented with NCBI Prokaryotic Genome Annotation Pipeline (PGAP) (Tatusova et al., 2016) and the following tools: ISfinder v. 2–2022-04-06 (Siguier et al., 2006), *PhiSpy* v.4.2.21 (Akhter et al., 2012), MobileElementFinder v1.0.3 (Johansson et al., 2020) and SPIFinder2 (Roer et al., 2016). For SPIFinder2, thresholds of 80 and 90% of minimum sequence coverage and identity, respectively, were used to identify *Salmonella* Pathogenicity Islands (SPIs). Visualization occurred with circos v.0.69–6 (Krzywinski et al., 2009).

### 2.7. Short-read sequencing of related *Salmonella* Agona isolates

For a representative selection of the *S.* Agona isolates received by the strain collection of the German NRL for *Salmonella*, isolates of different isolation sources, isolation years and geographic origins were sequenced (Supplementary File 6). Genomic DNA was prepared as described above and short-read libraries were prepared using the Nextera DNA Flex Library Prep Kit (Illumina, San Diego, CA, USA Illumina). Sequencing occurred on either a NextSeq 500 or a MiSeq device (Illumina, San Diego, CA, USA). MiSeq sequencing was performed as described for isolate 18-SA00377-0. For the NextSeq, paired-end sequencing was performed in 2 × 151 bp using the NextSeq 500/550 Mid Output Kit v2.5 (300 Cycles).

### 2.8. Sequence data, allele calling and visualization

Short-read data of 18-SA00377 is available in BioProject PRJNA706607 and was compared to genetically similar isolates on NCBI Pathogen Detection database (accessed on 12.12.2022 at^6^) based on Single Nucleotide Polymorphisms (SNPs). Within the SNP cluster, three closely related isolates were identified and included in the phylogenetic analysis.

Additionally, all available *S.* Agona sequences (157 isolates, excluding duplicates) from the NRL for *Salmonella* (BioProject PRJEB31846 and BioProject PRJNA742494) were included for phylogenetic comparisons as well as the following six isolates: one MDR *S.* Agona sequence isolated from a silver gull and five isolates from recent European *S.* Agona outbreaks (Supplementary File 6). Where required, read-based sequencing data was assembled with the Assembly-based QUality Assessment for Microbial Isolate Sequencing (AQUAMIS) pipeline (Deneke et al., 2021a). A *S.* Agona isolate (GCA_011632245.1) with a highly similar resistance profile to 18-SA00377 (more than 80% overlap of ARGs) was also included in the phylogenetic comparison. Thus, a total of 167 isolates were included in the phylogenetic analysis using the cgMLST workflow ChewieSnake (Deneke et al., 2021b). This workflow implements the allele calling software chewBBACA and generates an allele distance matrix, cluster membership, and phylogeny. The resulting allele distance matrix was visualized as a minimum spanning tree using iTOL (Letunic and Bork, 2021). Isolates were classified based on the number of different antimicrobial resistance classes conferred by their ARGs according to the comprehensive antibiotic resistance database (Alcock et al., 2019).

### 2.9. Average nucleotide identity (ANI)-based plasmid comparisons

The plasmid pSE18-SA00377-1 was analyzed using two tools in order to find closed plasmid sequences with high levels of similarity: the MOB-cluster tool from the MOB-suite (Robertson and Nash, 2018), which utilizes fast genomic distance estimation using Mash, and the web service COPLA (available at^7^), a plasmid classifying tool. The resulting plasmids deemed similar to pSE18-SA00377-1 by these two tools were characterized using BakCharak version 3.0.4 and their Average Nucleotide Identity (ANI) scores were calculated using FastANI (Jain et al., 2018). Plasmids with the highest ANI were mapped against pSE18-SA00377-1 using minimap2 version 2.22-r1101 (−asm5 option). The 11 plasmids with the highest mapping coverages against pSE18-SA00377-1 were visualized using circos v. 0.69–6 6 (Krzywinski et al., 2009). Easyfig v. 2.2 (Sullivan et al., 2011) was utilized to compare the structural organization of two subregional clusters of pSE18-SA00377-1 with four of these plasmids. Easyfig visualized the BLAST results from comparing the two clusters (100–146 kb and 220–260 kb) with the four plasmids with the highest mapping coverage (NC_012555, CP011601, CP042552, and NC_012556).

### 2.10. Querying of NCBI pathogen detection databases for high similarity ARG resistance profiles to *Salmonella* Agona isolate 18-SA00377

The AMR resistance profile of the 18-SA00377 isolate available on NCBI was compared to all available isolates of *S. enterica*, *E. coli* and *Shigella* sp., *Klebsiella pneumoniae*, *Enterobacter* sp., *Acinetobacter baumannii* on the respective NCBI Pathogen Detection databases (available at https://www.ncbi.nlm.nih.gov/pathogens)^8^ using a custom R script (Supplementary File 8) (databases download on 31.10.2022). The aim was to identify further *Salmonella* strains harboring plasmids that are similar to pSE18-SA00377-1, but for which no closed plasmid sequences were available. The draft genomes of all *Salmonella* genomes with a resistance profile that shared a minimum of 80% of the ARGs of 18-SA00377 were downloaded (Table 1), and mappings to pSE18-SA00377-1 with minimap2 version 2.22-r1101 (−asm5 option) (Li, 2018) were visualized using circos v. 0.69–6 (Krzywinski et al., 2009).

**Table 1:** Available metadata details of the nine *Salmonella* isolates mapped against pSE18-SA00377-1, covering their size in bp, date of collection, country of origin, isolation source, mapping coverage (%) to pSE-18-SA0037-1, assembly level and their accession numbers.

Color in Figure 6	Isolate	Collection year	Country of origin	Isolation source	Mapped coverage (%)	Assembly level	Accession number
	*Salmonella enterica* subsp. *enterica* serovar Agona	2018	Germany	Human (clinical)	94	Contig	GCA_020159705.1
	*Salmonella enterica* subsp. *enterica* serovar 4,[5],12:i:-	2015	USA	Animal (*Sus scrofa domesticus*)	93	Contig	GCA_007760705.1
	*Salmonella enterica*	NA	USA	Clinical	92	Contig	GCA_006389875.1
	*Salmonella enterica* subsp. *enterica* serovar 4,[5],12:i:-	2014	USA	Clinical (human)	92	Contig	GCA_006389855.1
	*Salmonella enterica*	2015	USA	Food (porcine)	92	Contig	GCA_005610165.1
	*Salmonella enterica* subsp. *enterica* serovar Agona	2019	USA	Animal (*Sus scrofa domesticus*)	92	Contig	GCA_011632245.1
	*Salmonella enterica* subsp. *enterica* serovar 4,12:i:-	2015	USA	Animal (*Sus scrofa*)	92	Contig	GCA_007756295.1
	*Salmonella enterica*	NA	NA	NA	89	Contig	GCA_011585645.1
	*Salmonella enterica*	2015	USA	Animal (*Bos taurus*)	88	Contig	GCA_005899425.1

### 2.11. Filter mating conjugation experiments

To analyze transferability of pSE18-SA00377-1, filter mating conjugation studies using sodium azide-resistant *E. coli* K-12 J53 recipient cells were conducted. Selective marker for p18-SA00377-1 was the *tetD* gene, located on the plasmid (see Table 2). Filter mating conjugation was performed as previously described (Hadziabdic et al., 2018). The reaction mixtures were plated on transconjugant selective LBA plates containing 12.5 mg/liter tetracycline (tetracycline, Sigma-Aldrich, Steinheim, Germany) and 100 mg/liter sodium azide (NaN_3_, Sigma-Aldrich, Darmstadt, Germany) and incubated at 37°C for approximately 42 h. A selection of potential transconjugants were picked and singulated on new selective tet/NaN_3_ plates. From these plates, single colonies were inoculated in LBL and incubated at 37°C under shaking condition (250 × rpm) for 16 h. Thermal cell lysis preparations were produced as previously described (Borowiak et al., 2017). Transconjugants were confirmed by *tetD* and J53 K12 screening PCR. The following primer pairs (with primer sequences in 5′-3′ direction and product length in parentheses) were used for the screening PCRs: For the J53 K12 screening K12R (ATCCTGCGCACCAATCAACAA; 1687 bp) and K12L (TTCCCACGGACATGAAGACTACA; 1687 bp) (Bauer et al., 2007), and for the *tet(D)* screening *tet(D)*-1 (AAACCATTACGGCATTCTGC; 787 bp) and *tet(D)*-2 (GACCGGATACACCATCCATC; 787 bp) (Ng et al., 2001).

**Table 2:** Breakdown of SPIs, plasmid markers, antibiotic resistance genes (ARGs), heavy metal resistance genes, and results from antibiotic susceptibility testing of the 18-SA00377 isolate including its four constituent plasmids.

Isolate	SPIs ^a)^ or Plasmid markers ^b)^	ARGs ^c) d)^ and amino acid substitution	Antibiotic susceptibility ^e)^	Heavy metal resistance gene ^c)^
SE18-SA00377-chromosome	SPI-1, SPI-2, SPI-4, SPI-8, SPI-9, and SPI-16	**Efflux transporter:** *mdsA* and *mdsB* **Fluoroquinolone:** *gyrA*_S83F **Fosfomycin:** *fosA7.6*	**Fluoroquinolone:** CIP =4 (S) and NAL >128 (R) **Fosfomycin:** FOS >4 **Aminoglycoside:** GEN >132 (R), KAN >64, and STR >32 **Beta-lactam:** AMP >64 (R), ETP < =0.015 (S), FEP =16 (R), FOT >4 (R), IMI =0.025, FOX =8 (S), PEN >2, MERO <=0.03 (S) and TAZ >8 (R) **Diaminopyrimidine:** TMP >32 (R) **Macrolide:** AZI =16 (S) **Peptide:** VAN >16 **Phenicol:** CHL >128 (R) **Polymyxins:** COL <= 1 (S) **Rifamycin:** RIF >0.5 **Sulfonamide** SMX >1,024 (R) **Tetracycline** TET >64 (R) and TGC =1 (S)	**Gold resistance:** *golS* and *golT*
pSE18-SA00377-1	RepA_1_pKPC-CAV1321, IncHI2A and IncHI2	**Aminoglycosides:** *aac(3)-Iig*, *aac(6′)-Iic*, *aadA2, aph(3′)-Ia*, *aph(3″)-Ib* and *aph(6)-Id* **Beta-lactam:** *bla*_SHV-12_ **Diaminopyrimidine:** *dfrA19* **Efflux transporter:** *qacEΔ1* * Macrolide: *ere(A)* * Peptide antibiotic: *mcr-9.1* **Phenicol:** *catA2* * Rifamycin: *arr* **Sulfonamide:** *sul1* * and *sul2* **Tetracycline:** *tet(D)*		**Arsenic resistance:** *arsB*, *arsC*, and *arsH* **Copper resistance:** *pcoE*, *pcoR*, and *pcoS* **Mercury resistance:** *merA*, *merD*, *merE*, and *merT* **Nickel/Cobalt resistance:** *rcnA* and *rcnR* **Tellurium resistance:** *terD*, *terW*, and *terZ*
pSE18-SA00377-2	p0111			
pSE18-SA00377-3	IncX3	**Aminoglycoside:** *aac(3)-IIe* **Beta-lactam:** *bla*_TEM-1_ **Fluoroquinolone:** *qnrS1* **Phenicol:** *floR*		
pSE18-SA00377-4	Col(Ye4449) and Col(MGD2)			

^a)^ SPIFinder2 (Roer et al., 2016) with minimum sequence coverage of 90% and minimum sequence identity of 80%, ^b)^ ABRicate (available at https://github.com/tseemann/abricate) and CGE PlasmidFinder (Carattoli et al., 2014), ^c)^ NCBI AMRFinder (Feldgarden et al., 2021) and ^d)^ Comprehensive Antibiotic Resistance Database (Alcock et al., 2019). ^e)^ For MIC testing the CLSI guidelines (version A7-M11) were followed and for interpretation the epidemiological cutoff values provided by EUCAST. Duplicates are marked with *.

The PCR reactions were prepared in 25 μL including 12.5 μL 2x DreamTaq Green PCR Master Mix (Thermo Scientific, Vilnius, Lithuania), 2.5 μL of each 10 μM primer dilution (s. above for details), 5.5 μL PCR grade water and 2 μL of thermal cell lysis preparation as template DNA and carried out as follows: initial denaturation for 5 min at 95°C, 30 cycles denaturation for 30 s at 95°C, primer annealing for 30 s at 54°C and elongation for 1 min [*tet(D)*] or 1:40 min (J53 K12) at 72°C followed by a final elongation step for 10 min at 72°C.

## 3. Results and discussion

### 3.1. Features of the 18-SA00377 chromosome and its plasmids

Illumina short-read and ONT long-read sequencing resulted in a hybrid assembly of one *Salmonella* chromosome with 4,856,956 bp and four plasmids: one IncHI2 plasmid (pSE18-SA00377-1) of 295,499 bp, one p0111 plasmid (pSE18-SA00377-2) of 94,574 bp, one IncX3 plasmid (pSE18-SA00377-3) of 50,931 bp and one Col (Ye4449) plasmid (pSE18-SA00377-4) of 5,284 bp. These five constituent assemblies of 18-SA00377 are available online on NCBI, grouped under the GenBank Accession number GCA_021497565.1 and BioProject PRJNA706607 with their individual Genbank Accession numbers as follows: CP071388.1 (chromosome), CP071389.1 (pSE18-SA00377-1), CP071390.1 (pSE18-SA00377-2), CP071391.1 (pSE18-SA00377-3), and CP071392.1 (pSE18-SA00377-4).

The presence of at least three plasmids was confirmed by PFGE using the S1 nuclease (Supplementary File 1). The sizes of these three plasmids were determined to be around 45, 80 and 320 kb. While varying slightly from the whole genome sequencing results this can be explained by the fact that S1-PFGE is more reliable at ascertaining plasmid sizes above 100 kb (Barton et al., 1995; Li et al., 2022) and can be unreliable for smaller plasmid sizes (Zhang et al., 2020; Juraschek et al., 2021).

Following genotypic characterization using the BakCharak pipeline and NCBI PGAP, the isolate 18-SA00377 was found to exhibit one DNA gyrase amino acid substitution at codon 83 (*gyrA_S83F*) and 23 different antibiotic resistance genes (ARGs). In total, the isolate 18-SA00377 carries 27 ARGs as four ARGs [*catA2*, *qacEΔ1*, *sul1* and *ere(A)*] are in duplicate and they confer resistance to 12 different classes of antibiotics resistance (Table 2). This was supported by antimicrobial susceptibility testing against antimicrobials of nearly every drug class, excluding efflux transporters. Moreover, the isolate harbors resistance genes against six heavy metals (gold, tellurium, arsenic, mercury, copper, and nickel/cobalt) as well as containing 131 chromosomal virulence factors belonging to seven classes (adherence, antimicrobial activity/competitive advantage, effector delivery system, immune modulation, invasion, nutritional/metabolic factor, and regulation) (data not shown). The isolate’s chromosome also harbors six *Salmonella* Pathogenicity Islands (SPIs) – SPI-1, SPI-2, SPI-4, SPI-8, SPI-9, and SPI-16 – with a sequence coverage of minimum 90% and sequence identity of above 80%. The following nine further SPIs were identified having a sequence coverage between 30 and 80%, but sequence identities of above 90%: SPI-3, SPI-12 (two copies), SPI-11, SPI-5, SPI-19, SPI-16 (two copies). Three of these SPIs (SPI-1, SPI-2, and SPI-4) have important roles in the virulence of *Salmonella* infections and can be found in all serovars of *Salmonella enterica*. Both SPI-1 and SPI-2 encode a distinct of type III protein secretion system (Jennings et al., 2017; Lou et al., 2019), which are, *inter alia*, important for the penetration and invasion of epithelial intestinal cells. On the other hand, the role of SPI-4 encodes a type I secretion system, which is crucial for adhesion during *Salmonella* infections (Gerlach et al., 2007). Two other SPIs – SPI-8 and SPI-9 – have been characterized based on the complete genome sequence of a *S.* Typhi CT18 strain (Parkhill et al., 2001). The function of SPI-8 is not well understood, but it has been shown to conferring resistance to bacteriocins (van Asten and van Dijk, 2005), while SPI-9, similar to SPI-4, encodes a type I secretion system as well as a RTX toxin-like protein (Velásquez et al., 2016). Lastly, SPI-16 has a role in immune evasion by carrying genes involved in O-antigen variation (Ilyas et al., 2017). Of the nine other SPIs present, the SPI-5 and SPI-3 are of particular importance as they are important mediators in host colonization and intracellular survival (Blanc-Potard et al., 1999), as well as the enteric stage of a *Salmonella* infections (Marcus et al., 2000), respectively. Overall, the presence of such a wide range of SPIs in a single isolate underlines the pathogenic potential of 18-SA00377. Thus, as previous outbreaks of *S*. Agona have shown, this serovar can cause human harm and the presence of both multidrug resistance and heavy metal resistances, highlight the importance of finding closely related isolates and establishing phylogeny.

### 3.2. Phylogenetic analysis of 18-SA00377

NCBI Pathogen Detection database based on Single Nucleotide Polymorphism (SNP) was searched for closely related isolates. This showed that the isolate 18-SA00377 is, with a minimum SNP distance of 31, only distantly related to three other *S.* Agona isolates: GCA_011585645.1, GCA_006296875, and GCA_020159705.1) (Supplementary File 6).

**Figure 1:**
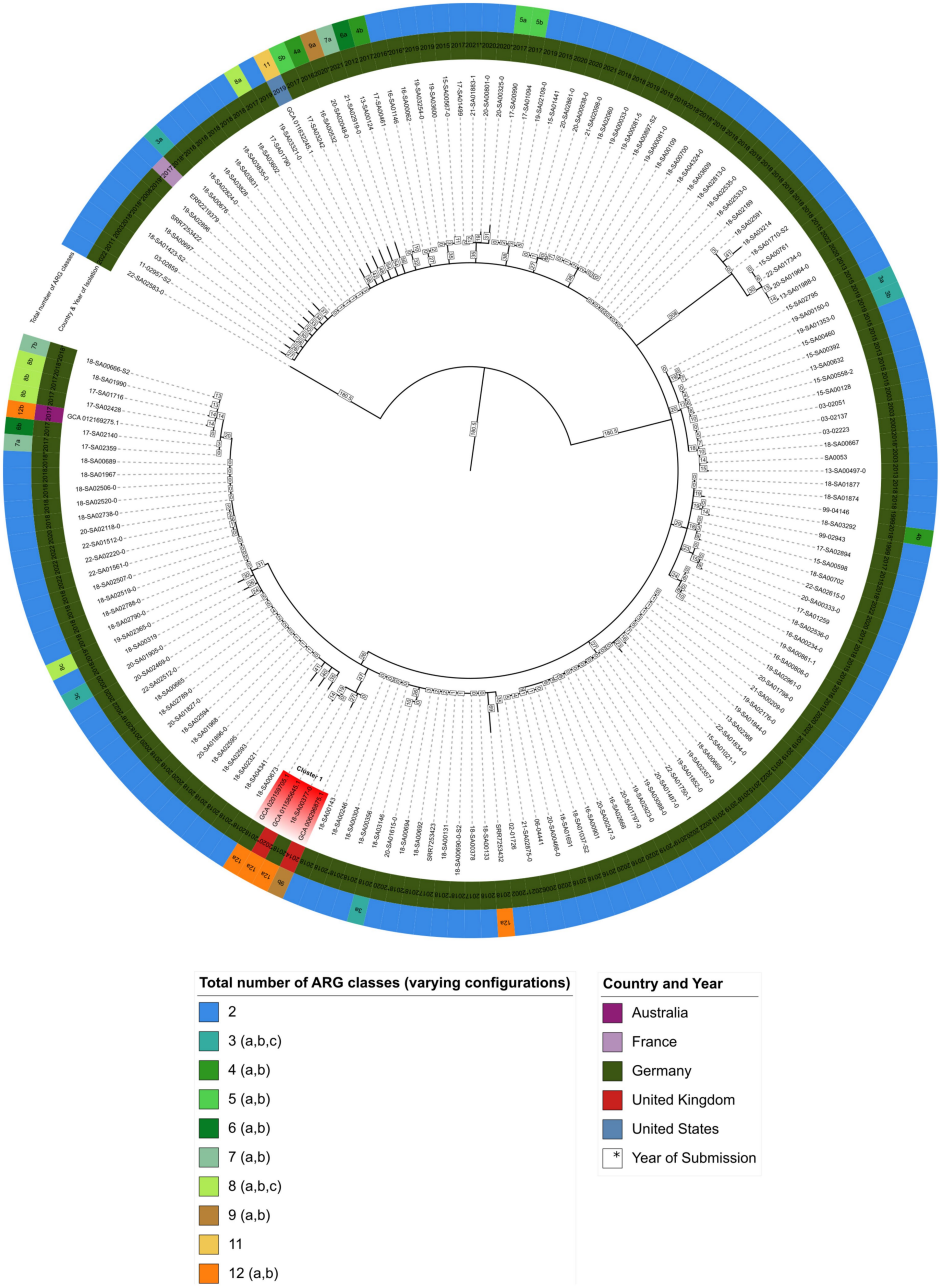
Minimum spanning tree showing hierarchical clustering between 167 *S.* Agona isolates. Inner circle indicates the country and isolation year, outer circle represents the number of ARG classes for each isolate, respectively (Supplementary File 6). Branch lengths in square boxes indicate minimum allelic distance between isolates. Varying configurations in the total number of different antibiotic resistance drug classes are indicated with a, b, and c – for a detailed breakdown see Supplementary File 7.

Next, all available sequences of *S.* Agona isolates from the German NRL for *Salmonella* was supplemented with *S.* Agona sequences from four other sources. Firstly, from three foodborne outbreaks: the 2017/18 infant formula outbreak in France (Jourdan-da Silva et al., 2018), the 2002/03 herbal tea outbreak (Koch et al., 2005), and the outbreak caused by a Bavarian feed product (Dangel et al., 2019). Secondly, three isolates from the aforementioned NCBI Pathogen Detection database (GCA_011585645.1, GCA_006296875, and GCA_020159705.1) as well as the sequence of a extensively drug-resistant *S.* Agona isolated from a silver gull (GCA_012169275.1) (Cummins et al., 2020). Lastly, a *S.* Agona isolate (GCA_011632245.1) with a high similarity ARG resistance profile was also included. A cgMLST analysis of these 167 *S.* Agona sequences (Supplementary File 6) was completed and visualized as a minimum spanning tree (Figure 1) (Letunic and Bork, 2021).

As shown in Figure 1, isolate 18-SA00377 is located within a clade, here named cluster A, with three other isolates: GCA_020159705.1, GCA_011585645.1 and GCA_006296875.1 with minimum allelic distances of 60, 19 and 27, respectively. These three isolates are not isolates from the German NRL for *Salmonella*, but were found via the NCBI Pathogen Detection database search. Two isolates, GCA_020159705.1 and GCA_006296875.1, were isolated from human sources in Germany in 2018 and the United Kingdom in 2014, respectively, while for GCA_011585645.1 metadata was not available. The closest *S.* Agona isolate to 18-SA00377 from the German NRL *Salmonella* has a minimum allelic distance of 74. The closest outbreak-associated *S.* Agona isolate is SRR7253423 from the Bavarian prevalence study, isolated from animal feed in Germany in 2017 (Dangel et al., 2019). As the allelic distances both between isolates within cluster A and to the closest cluster of isolates from the NRL for *Salmonella* exceed cut-offs previously used for defining distinct *Salmonella* outbreak clusters (Simon et al., 2018; Meinen et al., 2019), a recent common ancestor cannot be pinpointed by the cgMLST of 167 *S.* Agona isolates.

The total number of ARG drug classes across the 167 *S.* Agona isolates varies considerably and is not uniformly distributed. The majority of *S.* Agona isolates, including all the available *S.* Agona sequences in the NRL for *Salmonella*, harbor ARGs against only two classes, fosfomycin (*fosA7.2*) and efflux transporter subunits (*mdsA* and *mdsB*). Only a minority of *S.* Agona isolates (*n* = 8) carry resistance genes encoding for nine or more drug classes. Nevertheless, the four isolates in cluster A all carry ARGs against a minimum of nine antibiotic classes. Isolate 18-SA00377 is one of the most resistant isolates with 23 distinct ARGs conferring resistance to 12 different classes. There is an overlap between the antibiotic classes, with all four isolates in cluster A sharing resistances against the aforementioned fosfomycin and efflux transporter subunits, as well as the peptide antibiotic colistin (*mcr-9.1*), beta-lactams, fluoroquinolones, aminoglycosides, sulfonamides, tetracyclines, and diaminopyrimidine (see Supplementary Files 6, 7). Since the cgMLST analysis did not reveal a recent common ancestor for 18-SA00377, the antibiotic resistance profiles within cluster A and the unique resistance profile of 18-SA00377 highlight the importance of finding another potential source of the resistance properties of 18-SA00377, namely associated mobile genetic elements.

### 3.3. Plasmid descriptions and comparisons

Next, we focused to finding isolates with similar antibacterial resistance profiles in a wider set of genera of *Enterobacteriaceae*. As the majority of its unique MDR resistance profile is due to the ARGs carried on the isolate’s largest plasmid, pSE18-SA00377-1, the plasmid was characterized. Assembly of the plasmids was possible by combining long-read and short-read sequencing data. Annotation of the assembly revealed that pSE18-SA00377-1 harbors a total of 20 ARGs (Table 2), including two copies of each of *catA2*, *qacEΔ1, sul1*, and *ere(A)*. A further four ARGs (*qnrS1, bla*_TEM-1_, *aac(3)-IIe*, and *floR*) are located on pSE18-SA00377-3 and each associated with a putative composite transposon of the IS*6* family (Supplementary File 4). The plasmids, pSE18-SA00377-2 and pSE18-SA00377-4, do not carry any antibiotic resistance genes, but pSE18-SA00377-4 carries two replicons, Col(MGD2) and Col(Ye4449) as well as mobilization genes (*mobC*, *mbeD*, *mbeB*, *mbA*) (Supplementary File 5).

Similar to the ARG distribution, the distribution of genes conferring heavy metal resistances is skewed toward pSE18-SA00377-1, where genes encoding against six different heavy metal resistances are located (Table 2). While the genome only carries *golS* and *golT* genes, encoding for gold resistance, the resistance genes against arsenic (*arsB, arsC*, and *arsH*), copper (*pcoE, pcoR, pcoS)*, mercury (*merA, merD, merE*), nickel/cobalt (*rcnA* and *rcnR)*, and tellurium (*terD, terW, terZ*) all are located on pSE18-SA00377-1.

The makeup of pSE18-SA00377-1 is not only limited to a large number of resistance genes, it also carries three plasmid markers: RepA_1_pKPC-CAV1321 as well as IncHI2A and IncHI2. Moreover, it also carries genes for the purpose of conjugational transfer by encoding for numerous conjugal transfer proteins (e.g., *traK, traB, traV,* etc.) as well as an *oriT and repB* replication initiator. The presence of these genes supports the assumption that the pSE18-SA00377-1 plasmid is transferrable *in vivo*.

Transmissibility of pSE18-SA00377-1 by conjugation was confirmed experimentally by *in vitro* filter mating experiments. Successful transfer of the plasmid to the recipient, *E. coli* J53 K12, occurred, supporting the bioinformatic analysis that pSE18-SA00377-1 is conjugative. Furthermore, as the recipient *E. coli* K12 J53 belongs to a different genus of the family *Enterobacteriaceae*, this supports the subsequent bioinformatic analyses that indicates that large parts of the pSE18-SA00377-1 plasmid backbone can be found in other bacterial genera and that the host range of the pSE18-SA00377-1 plasmid is not limited to *Salmonella*.

**Figure 2:**
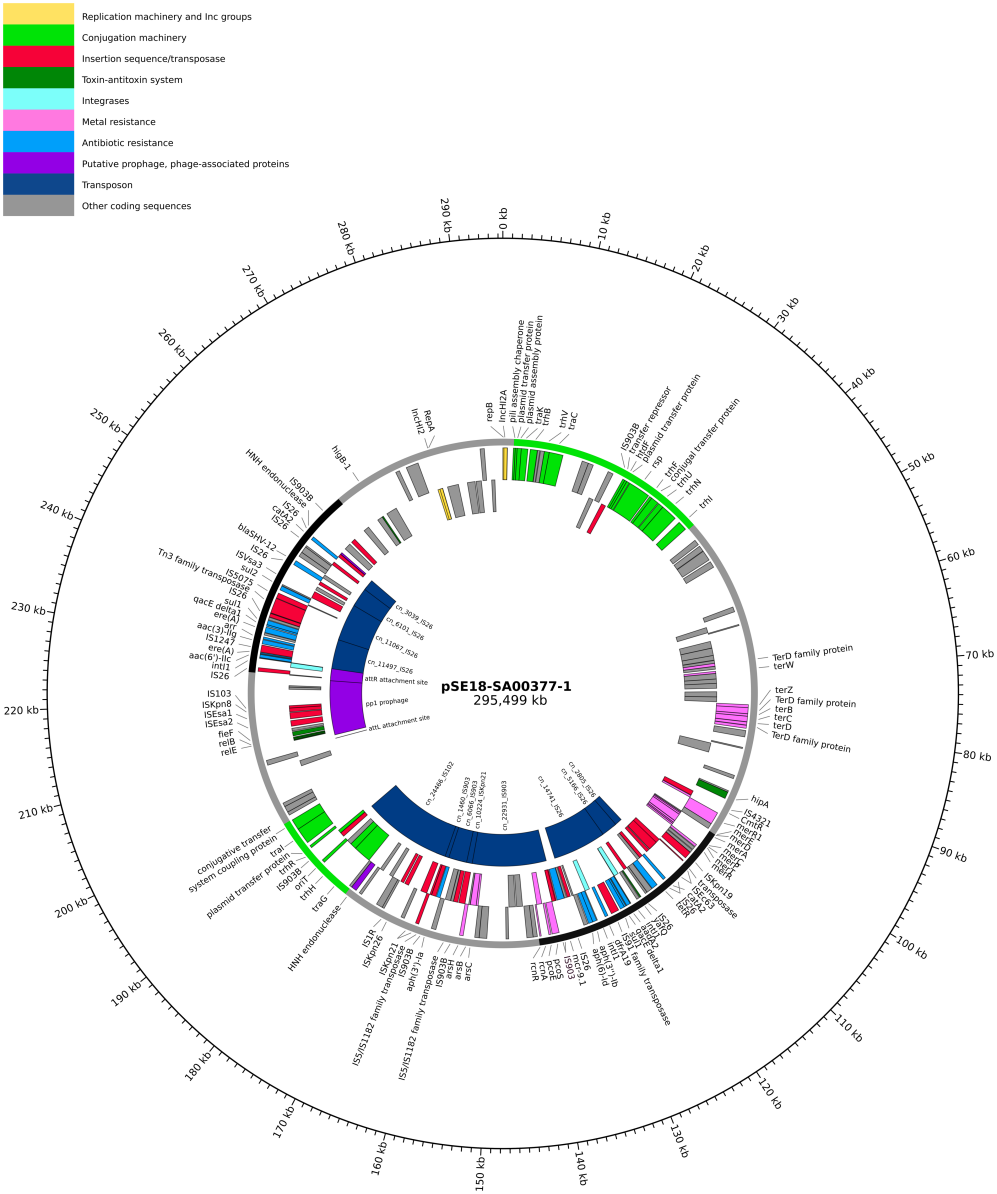
Visualization of the pSE18-SA00377-1 plasmid. Annotations are colored according to nine categories: replication machinery and incompatibility group in yellow, conjugation machinery in light green, insertion sequences and transposases in red, toxin-antitoxin system in dark green, integrases in turquoise, heavy metal resistances in pink, and antibiotic resistances in light blue. On the innermost track, the putative prophage and prophage-associated proteins are shown in purple, while putative transposons are colored dark blue. Coding sequences for other gene products are colored in gray. The outermost ring shows the plasmid backbone in gray and highlights regions of interest: cluster 1 (95–140 kb) and cluster 2 (230–250 kb) in black and regions with conjugation machinery in green (0–40 kb and 180–200 kb). For a complete list of available annotations, see Supplementary File 3.

Arrangement of ARGs, heavy metal resistances, conjugation machinery and transposable elements on pSE18-SA00377-1 is concentrated within two distinct clusters, as evident in the visualization of the plasmid’s annotations (Figure 2). The annotations of the remaining plasmids, pSE18-SA00377-2, pSE18-SA00377-3, and pSE18-SA00377-4, were not visualized, but their consensus annotations are available as Supplementary Files 3–5, respectively. The first cluster on pSE18-SA00377-1, ~ 95–140 kb, contains nine ARGs interspersed with transposases. Four ARGs – *aadA2*, *qacEΔ1*, *sul1*, and *dfrA19* – are part of a class 1 integron cassette as they are flanked on both sides by class 1 integron integrases (*intI1*) and have Pc and PintI1 promoters in their vicinity as well as two recombination crossover points (*attC* and *attI*). Moreover, all nine ARGs in this region are associated with putative composite transposons. Similarly, six ARGs – *aadA2*, *qacEΔ1*, *sul1, dfrA19, aph(3″)-Ib* and *aph(6)-Id* – are associated with a single putative composite transposon, cn_14741_IS26, which is flanked by two IS*26* elements. A similar picture emerges for heavy metal resistances, with genes encoding for copper, nickel/cobalt, and nickel resistances associated with a putative composite transposon, cn_22931_IS903.

A second cluster with a high concentration of ARGs spans the range 230–250 kb and contains further 10 ARGs. The *aac(6′)-IIc* gene is associated with an incomplete class 1 integron as a class 1 integron integrase (*intI1*), Pc and PintI1 promoters and recombination crossover points (*attC* and *attI*) are in its vicinity. Similar to the first cluster, all ARGs are located on putative composite transposons, with the first seven ARGs being associated with the cn_11497_IS26 putative composite transposon, while each of the remaining three ARGs are associated with a composite transposons of the IS*6* family. Between these two distinct regions of the plasmid, pSE18-SA00377-1 also encodes components for a type II toxin-antitoxin system (*relE*, *relB*) and an iron efflux transporter (*fieF*).

Closer analysis of the plasmid’s genetic makeup revealed that this large plasmid has two distinct regions of densely clustered ARGs, which are associated with putative composite transposons. This, in conjunction with the presence of numerous copies of highly active IS*26* elements, that frequently mediate recombination in *Salmonella* spp. (Doublet et al., 2009), suggests that this plasmid has a complex evolution with frequent insertions of ARGs.

### 3.4. Comparative analysis of the pSE18-SA00377-1 plasmid

**Figure 3:**
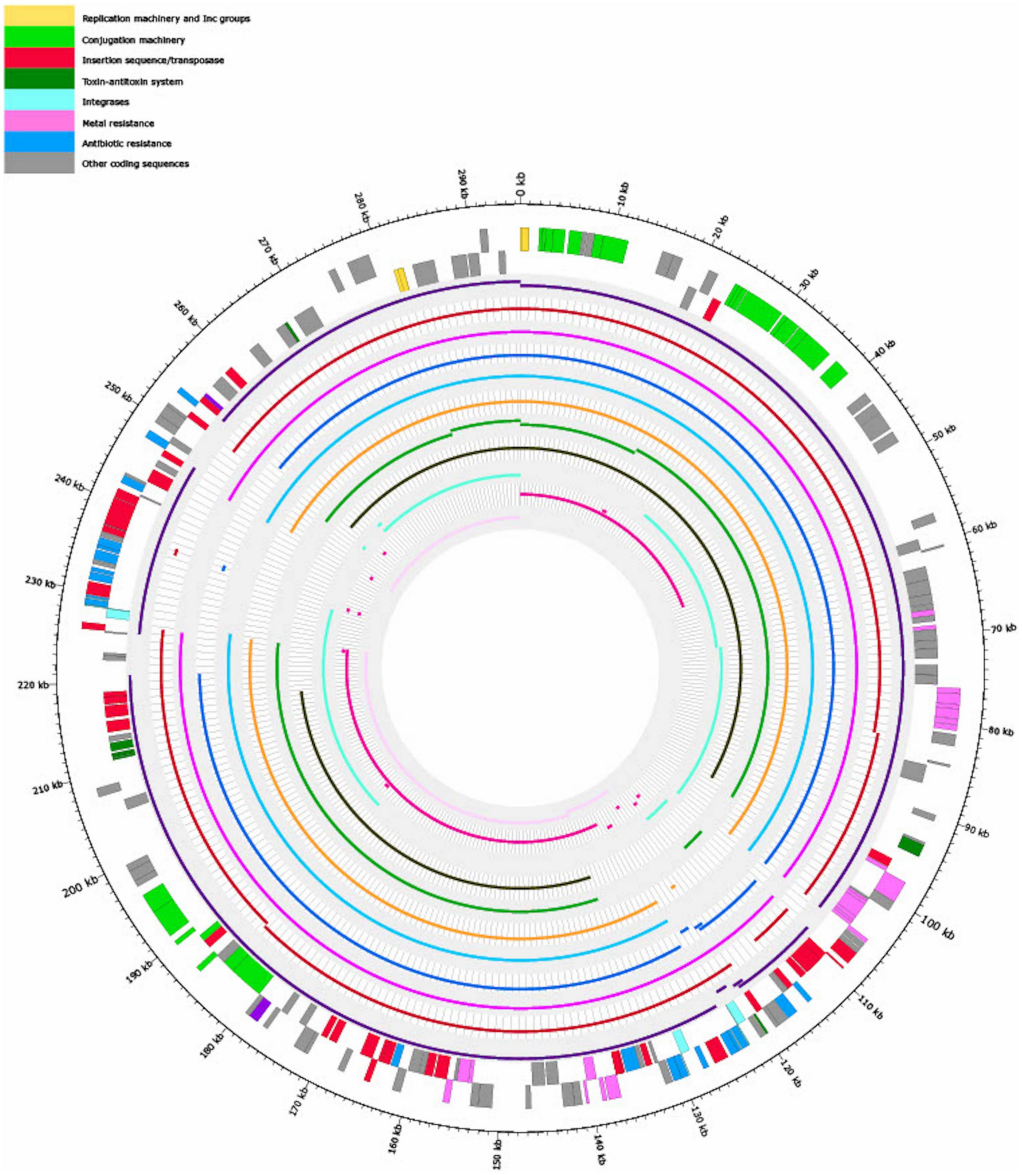
Visualization of mapping results of 11 plasmids against pSE18-SA00377-1 (outermost two tracks), in order of decreasing mapping coverage (%), with lowest coverage of the *Citrobacter farmeri* plasmid (light pink) and highest coverage of the *Enterobacter cloacae* plasmid pEC-IMP (dark purple) (see Table 3). Regions of the 11 plasmids that did not map to pSE18-SA00377-1 are not shown. The outermost black circle designates the base positions around the plasmid. Farthest two tracks on the outside represents the pSE18-SA00377-1 plasmid annotations, with replication machinery and incompatability groups colored in yellow, conjugation machinery in light green, insertion sequences and transposases in red, toxin-antitoxin system in dark green, integrases in turquoise, heavy metal resistances in pink, and antibiotic resistances in light blue. Coding sequences for other gene products are colored in gray. For labeling of annotations refer to Figure 2.

In order to find closely related plasmids of pSE18-SA00377-1, the outputs of two tools, the MOB-cluster tool and the COPLA web tool, were further analyzed by calculating their ANI scores. Consequent ranking by ANI score and matching antibiotic resistance profiles yielded 11 high-similarity plasmids (Table 3), which were mapped to pSE18-SA00377-1 (Figure 3).

**Table 3:** Metadata details of the 11 plasmids mapped against pSE18-SA00377-1, covering their size in bp, date of collection or NCBI submission, country of origin, category of isolation source, percentage of coverage when mapped to pSE-18-SA0037-1, and their accession.

Color in Figure 2	Isolate	Size (bp)	Collection year	Country of origin	Isolation source	Mapped coverage (%)	Accession number
	*Enterobacter cloacae* plasmid pEC-IMP	318,782	2008 ^a)^	Taiwan	Clinical	92	NC_012555
	*Phytobacter ursingii* strain CAV1151 plasmid pCAV1151-296	295,619	2009	USA	Clinical (human)	89	CP011601.1
	*Enterobacter hormaechei* strain C45 plasmid pC45_001	288,659	2013	Australia	Clinical (human)	85	CP042552
	*Enterobacter cloacae* plasmid pEC-IMPQ	324,503	2008 ^a)^	Taiwan	Clinical	85	NC_012556
	*Enterobacter asburiae* strain AMA 497 plasmid pOXA436	314,137	2014	Denmark	Clinical (human urine)	85	KY863418
	*Enterobacter hormaechei* strain C15117 plasmid pSPRC-Echo1	339,920	2007	Australia	Clinical (Burns Unit Surveillance)	85	CP032842
	*S.* Typhimurium strain MU1 plasmid pIMP4-SEM1	339,962	2016	Australia	Animal (cat)	79	KX810825
	*Enterobacter hormaechei* strain MS7884A plasmid pMS7884A	330,060	2015	Australia	Clinical (human)	71	CP022533.1
	*S.* Heidelberg strain 09–036813-1A plasmid p09-036813-1A_261	261,310	2009	Canada	Animal (horse)	57	CP016526.1
	*Enterobacter hormaechei* subsp. *steigerwaltii* strain 34,977 plasmid p34977-263.138 kb	263,138	2009	USA	Clinical (human)	55	CP012170.1
	*Citrobacter farmeri* strain AUSMDU00008141 plasmid pAUSMDU8141-1	328,945	2015	Australia	Clinical (human)	51	CP022696.1

^a)^ Year of submission to NCBI.

When mapped against pSE18-SA00377-1, these 11 plasmids exhibit a nucleotide coverage exceeding 50%. They were isolated from a wide range of bacterial isolates and their geographical origins span Australia (CP022696.1, CP022533.1, CP042552, CP032842, and KX810825), Taiwan (NC_012555 and NC_012556), the United States (CP012170.1 and CP011601.1), Canada (CP016526.1), and Denmark (KY863418). Furthermore, these plasmids were mostly isolated from *Enterobacter* species in a clinical context, but were also found in *Salmonella*, *Citrobacter*, and *Phytobacter*. The *Enterobacter* and *Citrobacter* plasmids were found exclusively in clinical isolates, while the *Salmonella* plasmids were isolated from animals. Particularly, the occurrence of the *Salmonella* plasmid, KX810825, is of concern as it was found in a companion animal (Abraham et al., 2016).

Figure 3 shows that the coverages against pSE18-SA00377-1 (*E. cloacae* pEC-IMP, *Phytobacter ursingii*, *Enterobacter hormaechei*, and *E. cloacae* pEC-IMPQ) all map in the region of the first cluster of ARGs. However, for the second cluster of ARGs at 230–250 kb only the *E. cloacae* pEC-IMP plasmid (in dark purple) maps against large parts of this cluster, while the other six plasmids do not. All eleven plasmids wholly map against the majority of the pSE18-SA00377-1 plasmid backbone, including the conjugation and replication machinery.

### 3.5. Structural representation of two pSE18-SA00377-1 subregions

**Figure 4:**
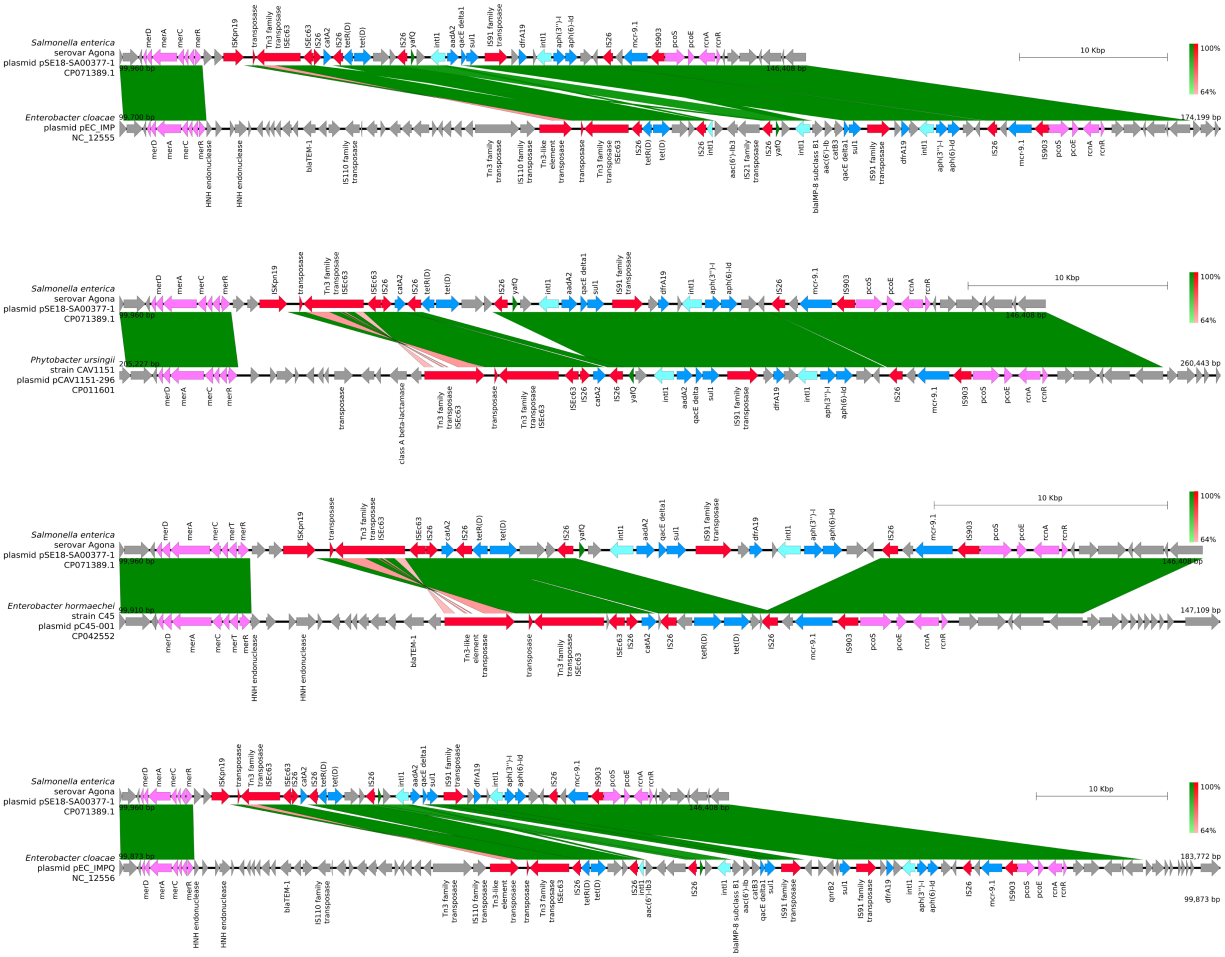
Visualization of comparison of the structural features between the 100–146 kb region of pSE18-SA00377-1 and four plasmids with the highest nucleotide coverages (NC_012555, CP011601, CP042552, and NC_012556). Annotations of coding sequences are only colored if BLAST results indicated homology between pSE18-SA00377-1 and the other plasmid. Annotation coloring is based on the same eight categories as in Figures 2, 3, with insertion sequences and transposases in red, toxin-antitoxin system in dark green, integrases in turquoise, heavy metal resistances in pink, and antibiotic resistances in light blue. Coding sequences present in the plasmids belonging to one of the eight colored annotation categories, but showing no homology by blast to pSE18-SA00377-1, were labeled but left in gray.

For closer comparison of the organization of the ARGs and other features within the two aforementioned ARG clusters of pSE18-SA00377-1, these regions were compared to the 11 high-similarity plasmids using BLAST and visualized using easyfig. This closer inspection revealed that the organization of the first cluster of pSE18-SA00377-1, 100–146 kb, is conserved across four plasmids NC_012555, CP011601, CP042552, and NC_012556 (Figure 4).

All four of these plasmids harbor minimum eight of the nine ARGs constituent of this region, *catA2*, *tetD*, *aadA2*, *qacEΔ1*, *sul1*, *dfrA19*, *aph(3″)-Ib*, *aph(6)-Id*, and *mcr-9.1*. In two plasmids, CP016526 and CP012170, the region with its nine ARGs is present but in an inverted state and only partially. Four ARGs are located toward the end of the region, *dfrA19*, *aph(3″)-Ib*, *aph(6)-Id*, and *mcr-9.1*. They are present in both plasmids, while the remaining ARGs, *catA2*, *tetD*, *aadA2*, *qacEΔ1*, *sul1* have merged with the second cluster of ARGs (220–260 kb). In the five remaining plasmids, the organization of the ARGs is considerably changed, with the order of the ARGs split or shuffled (CP022696, CP022533, CP042552, CP032842, and KX810825) or merged partially (CP042552) with the second cluster of ARGs.

**Figure 5:**
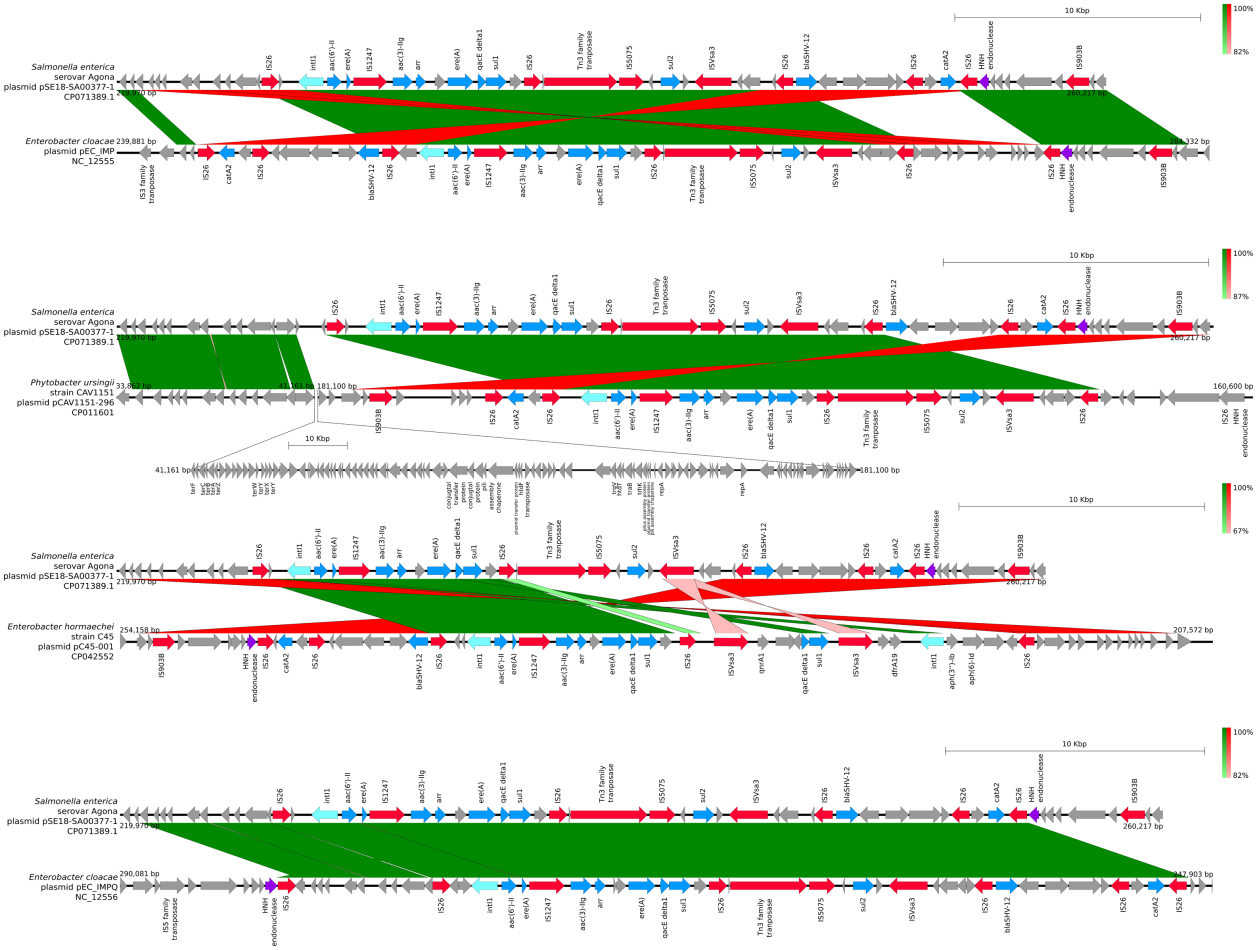
Visualization of comparison of the structural features between the 220–260 kb region of pSE18-SA00377-1 and four plasmids with the highest nucleotide coverages (NC_012555, CP011601, CP042552, and NC_012556). Annotations of coding sequences are only colored if blast results indicated homology between pSE18-SA00377-1 and the other plasmid’s. Annotation coloring is based on the same eight categories as in Figures 2, 3, with insertion sequences and transposases in red, toxin-antitoxin system in dark green, integrases in turquoise, heavy metal resistances in pink, and antibiotic resistances in light blue. Coding sequences present in the plasmids belonging to one of the eight colored annotation categories, but showing no homology by blast to pSE18-SA00377-1, were labeled but left in gray.

Similar to the first region, the second cluster of ARGs at 220–260 kb of pSE18-SA00377-1 was compared to four of the 11 aforementioned high-similarity plasmids and visualized using easyfig (Figure 5). For this second cluster, two plasmids (NC_012555 and NC_012556) harbor the same structural features in the same organizational matter as pSE18-SA00377-1, while the remainder of the 11 plasmids only carry a partial ARG load.

The mapping of these 11 plasmids to pSE18-SA00377-1 indicates that the pSE18-SA00377-1 contains a conserved plasmid backbone which frequently occurs in other plasmids of other genera. However, the widespread geographical origin of these 11 plasmids and their occurrence in a wide range of bacterial genera does not allow for ascertaining a potential common origin. Nevertheless, the high number of ARGs as well as their associations with composite transposons indicates that multiple insertion events had occurred and led to this accumulation of resistance genes in two distinct clusters. This accumulation of resistance genes is also present in the other 11 plasmids, although the first cluster of ARGs seems to be more stable, being present in its entirety and same organizational structure in four other plasmids.

This analysis showed that plasmids with high nucleotide similarity to pSE18-SA00377-1 and similar antibiotic resistance profiles can be found. However, no plasmid with a mapping coverage of >80% had been isolated from *S.* Agona isolates, with the highest mapping coverages all belonging to plasmids isolated either from *Enterobacter* or *Phytobacter* isolates. As high-similarity plasmids to pSE18-SA00377-1 seem to be found in a wide variety of genera and different geographical origins, it can be speculated that pSE18-SA00377-1 had been taken up from other sources, potentially in a clinical environment as a majority of the closely-related plasmids were isolated from clinical sources. Alternatively, the pSE18-SA00377-1 plasmid could have been taken up from environmental sources, for example when wastewater is re-used for irrigation in agriculture, as the SE18-SA00377 isolate was isolated from a dietary supplement consisting of plant-based ingredients. These hypotheses are also supported by the potential origins of the other constituent plasmids of the 18-SA00377 isolate. Firstly, the second largest plasmid, pSE18-SA00377-2 (94,574 bp), is a P1-like phage plasmid, carrying the p0111 plasmid replication gene, which was first identified from an enterohemorrhagic *E. coli* strain (Ogura et al., 2009) and is still frequently found in *E. coli* isolates, including clinical and food isolates (Balbuena-Alonso et al., 2022). Moreover, it carries two prophage-like elements pp1 and pp2, which were first identified in the core genome of *E. faecalis* isolates (Matos et al., 2013).

As an IncX3 plasmid and carrier of the *bla*_TEM-1_ gene, the second smallest plasmid, pSE18-SA00377-3 (50,931 bp), plays a role in the dissemination of carbapenemase resistance genes. The IncX plasmid family has been reported in a wide variety of *Enterobacteriaceae* from different sources (Guo et al., 2022) and thus the plasmid is a cause of concern due to its additional ARG load of *aac(3)-IIe*, *qnrS1*, and *floR*. Furthermore, pSE18-SA00377-3 also carries *tmrB*, a gene encoding the tunicamycin resistance protein which confers resistance to tunicamycin in *Bacillus subtilis* (Noda et al., 1992).

### 3.6. Plasmids with high similarity antibiotic resistance profiles to 18-SA00377

In order to limit the search to closely related isolates of *Salmonella* but also other members of the *Enterobacteriaceae* family, several NCBI Pathogen Detection databases were queried for isolates with highly similar antibiotic resistance profiles (80% overlap in ARGs with 18-SA00377). This resulted in short-read sequences from the following four databases: *S. enterica*, *E. coli* and *Shigella*, *Klebsiella*, and *Citrobacter* (Table 4).

**Table 4:** Table showing the overview of isolates resulting from querying the respective NCBI Pathogen Detection databases with custom R script.

Isolates’ species	Countries of origin ^a)^	Years of collection	Isolation source ^a)^	Median mapped coverage (%)	Assembly levels	Median % of matching AMR genes	NCBI Pathogen detection database
*Salmonella enterica*	USA (7), Germany (1), NA (1)	2014–2019	environmental/other (5), clinical (3), NA (1)	92	All contigs	83	*Salmonella enterica*
*Klebsiella* ssp.	Montenegro (4), Canada (4), USA (3), Romania/Taiwan/France/Mexico/Australia (all 1)	2001–2021	Clinical (13), environmental/other (2)	91	All contigs	61	*Klebsiella pneumoniae*
*Escherichia coli*	USA (11), Russia (3), Germany/France (2), Australia/Canada/Chile/China/Czech Republic/Rwanda (all 1)	2007–2021	Clinical (11), environmental/other (7)	90	Contigs (19), scaffolds (5)	61	*E.coli* and *Shigella*
*Citrobacter* ssp.	USA (4), Australia (3), Canada (2), United Kingdom/China (both 1)	2010–2021	Clinical (11), environmental/other (1)	89	All contigs	61	*Citrobacter freundii*

^a)^ Numbers in brackets indicate the number of occurrences, NA, data not available.

**Figure 6:**
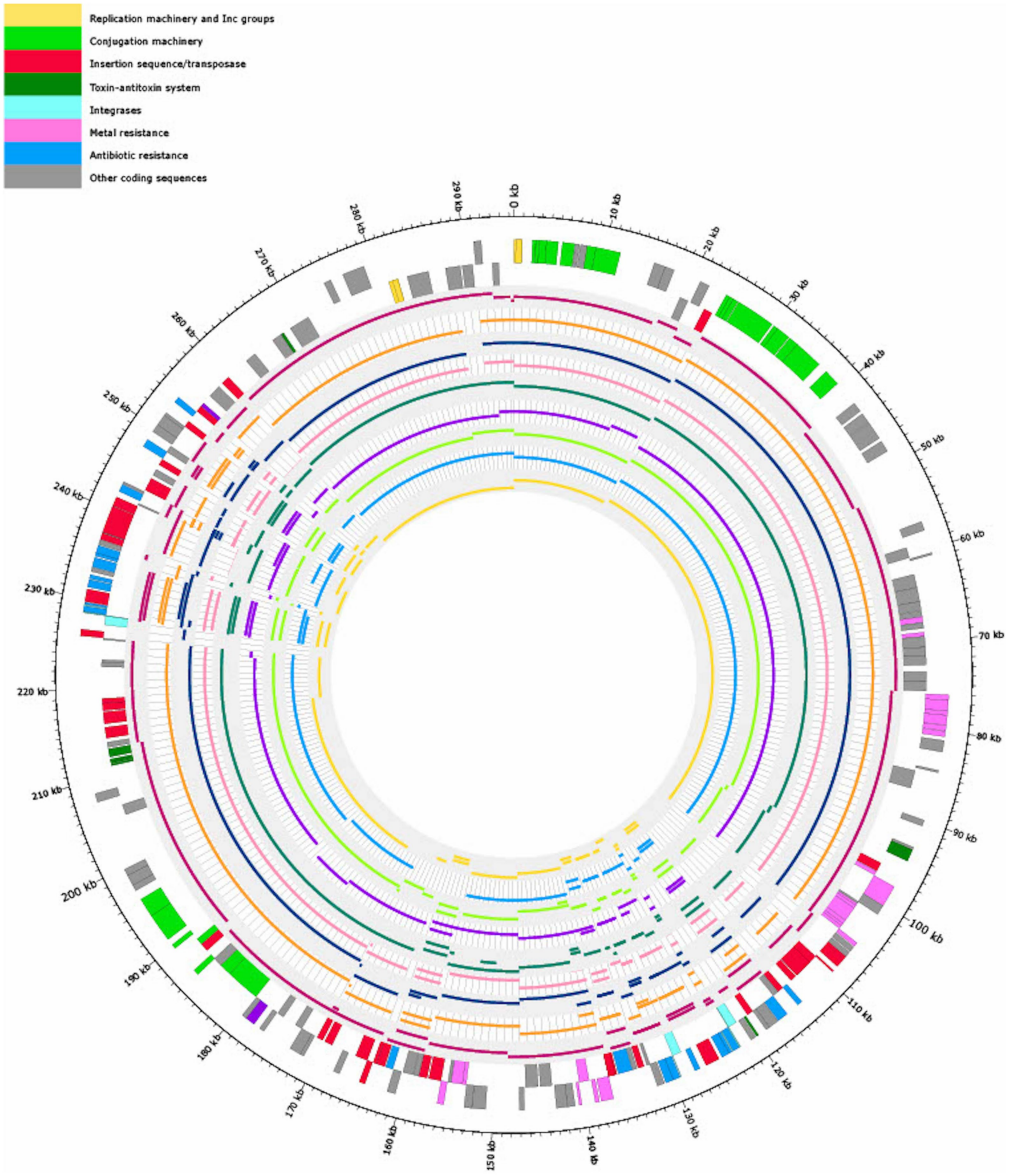
Visualization of mapping the contigs of nine *Salmonella* isolates (Table 1) with similar antibiotic resistance profiles (80% overlap of ARGs with 18-SA00377) to pSE18-SA00377-1. Regions of the nine *Salmonella* isolates that did not map to pSE18-SA00377-1 are not shown. The outermost black circle designates the base positions around the plasmid. Farthest two tracks on the outside represents the pSE18-SA00377-1 plasmid annotations, with replication machinery and incompatibility groups colored in yellow, conjugation machinery in light green, insertion sequences and transposases in red, toxin-antitoxin system in dark green, integrases in turquoise, heavy metal resistances in pink, and antibiotic resistances in light blue. Coding sequences for other gene products are colored in gray. For labeling of annotations refer to Figure 2.

The nine *Salmonella* isolates of different serovars, geographical origins, and isolation sources (Table 1) were compared by mapping of contigs to p18-SA00377-1 and mapping results visualized (Figure 6).

Four isolates were isolated from animal sources, three isolates from clinical sources, and one isolate from a porcine food source. The animal samples were all isolated from the United States, but their isolation types range from domestic pigs (*Sus scrofa domesticus*) to wild boar (*Sus scrofa*) and cattle (*Bos taurus*). However, these environmental samples harboring these plasmids were either of serovar Agona or 4,12:i:-, the monophasic variant of *S.* Typhimurium.

However, based on available isolates’ metadata, it was impossible to infer if the mapping against the pSE18-SA00377-1 occurred within their chromosomes or in constituent plasmids, due to draft character of the used short-read derived assemblies. Nevertheless, based on the numerous breaks in coverage of mapped sections and the large number of very small mapped sections (e.g., IS*26*), it might be that the ARGs are located in the isolates’ chromosomes and not on a plasmid and the two described ARG clusters. Furthermore, multiple occurrences of these areas could become merged into repetitive regions during assembly of the short-read data.

In conclusion, this SE18-SA00377 isolate belongs to a sublineage of *S. enterica* serovar Agona that is multidrug-resistant and might be plant-associated. Along with its four plasmids, pSE18-SA00377-1, pSE18-SA00377-2, pSE18-SA00377-3, and pSE18-SA00377-4, the isolate carries a total of 23 different ARGs, conferring resistance to 12 different classes of antibiotics, with its largest plasmid of 295,499 kb in size, pSE18-SA00377-1, conferring the majority of them. Moreover, the pSE18-SA00377-1 plasmid is not only the main carrier of antibiotic resistance genes but also of heavy metal resistances. The structure of this plasmid is striking as its ARGs have accumulated in two distinct regions. This accumulation of ARGs as well as the presence of these clusters and a large part of its backbone in plasmids isolated from a wide range of genera, matrices, years of isolation and geographical origins suggest that this plasmid has a complex history with numerous transmission events.

Further analysis of plasmids from human, veterinary, and environmental sources may provide further insights into the evolution of this plasmid. In particular, due to the highly drug-resistant nature of this plasmid, identifying potential reservoirs of multidrug-resistant isolates is crucial, as they have the capacity to disseminate antibiotic and metal resistance genes.

Here, we present an in-depth characterization of a multidrug-resistant *S.* Agona, isolated from dietary supplements in 2018. Its phylogeny to other *S.* Agona isolates from Germany was established and supplemented with available sequences of *S*. Agona that have been reported globally and are available in the NCBI database. Detailed annotation of its largest constituent plasmid included antimicrobial resistance genes on mobile genetic elements. Closely related plasmids were queried through a two-pronged approach: MOB-typing and taxonomic classification of plasmids. Lastly, structural comparisons with high-similarity plasmids revealed a composite plasmid structure, found in isolates from numerous other genera, geographic origins and isolation matrices. These analyses showed that this plasmid is a potential reservoir for antimicrobial and heavy metal resistance determinants and has the potential to adapt to various hosts and environments. Thus, highlighting the need for continued surveillance to prevent future outbreaks.

## Data availability statement

The datasets presented in this study can be found in online repositories. The names of the repository/repositories and accession number(s) can be found in the article/Supplementary material.

## Author contributions

LB: Formal analysis, Investigation, Methodology, Visualization, Writing – review & editing. MB: Formal analysis, Investigation, Methodology, Visualization, Writing – review & editing. CD: Data curation, Formal analysis, Investigation, Software, Writing – review & editing. JG: Conceptualization, Supervision, Writing – review & editing, Software. J-AH: Methodology, Writing – review & editing. BM: Project administration, Resources, Supervision, Writing – review & editing, Funding acquisition. IS: Project administration, Resources, Writing – review & editing. TA: Project administration, Supervision, Writing – review & editing. KN: Resources, Writing – review & editing. JF: Conceptualization, Investigation, Methodology, Project administration, Resources, Supervision, Writing – original draft, Writing – review & editing, Data curation, Formal analysis, Funding acquisition, Visualization.

## Footnotes

^1^ https://www.cdc.gov/pulsenet/pdf/ecoli-shigella-salmonella-pfge-protocol-508c.pdf

^2^ https://community.nanoporetech.com

^3^ https://github.com/rrwick/Porechop

^4^ https://gitlab.com/bfr_bioinformatics/bakcharak

^5^ https://github.com/tseemann/abricate

^6^ https://www.ncbi.nlm.nih.gov/pathogens

^7^ https://castillo.dicom.unican.es/copla

^8^ https://www.ncbi.nlm.nih.gov/pathogens/
