# Supplementary material for: Genetic characterization of a multidrug-resistant Salmonella enterica serovar Agona isolated from a dietary supplement in Germany
Source: Front Microbiol. 2023 Nov 15;14:1284929. doi: 10.3389/fmicb.2023.1284929 (PMC10686068; doi:10.3389/fmicb.2023.1284929)
Supplement: Supplementary file 1 [file Data_Sheet_1.zip › 1284929_Fischer_Data_Sheet_1.PDF]

```
# search for Enterobacteriaecaea isolates with highly similar AMR profiles in
NCBI Pathogen Detection database

#install dply library
library(dplyr)

# functions
compare_profiles <- function(amrmetadata_highres, query_profile){

  profile_comparison <-
do.call(rbind,apply(amrmetadata_highres,1,function(myreference){

    myreference_profile <- myreference["AMR_genotypes"] # selct single reference
    myreference_profile <- gsub('\"',"",myreference_profile) # clean
    myreference_profile_split <- strsplit(myreference_profile,",")[[1]]
    myreference_profile_split <- sapply(myreference_profile_split, function(x)
strsplit(x,"=")[[1]][1])

    myintersection <- intersect(myreference_profile_split,query_profile)
    myintersection_length <- length(myintersection)
    myintersection_percentage <- length(myintersection)/query_profile_count
    myintersection_genes <- paste(myintersection,collapse = ";")

    # additional genes
    mysetdiff <- setdiff(myreference_profile_split,query_profile)
    mysetdiff_count <- length(mysetdiff)
    mysetdiff_genes <- paste(mysetdiff,collapse = ";")

    # missing genes
    missing <- setdiff(query_profile,myreference_profile_split)
    missing_count <- length(missing)
    missing_genes <- paste(missing,collapse = ";")


    mysummary <- data.frame(
      biosample_acc = myreference["biosample_acc"],
      matching_genes_relative = myintersection_percentage,
      matching_genes_count = myintersection_length,
      matching_genes = myintersection_genes,
      additional_gene_count = mysetdiff_count,
      additional_genes = mysetdiff_genes,
      missing_count = missing_count,
      missing_genes = missing_genes,
      epi_type = myreference["epi_type"],
      geo_loc_name = myreference["geo_loc_name"],
      isolation_source = myreference["isolation_source"],
      serovar = myreference["serovar"]
    )

  }))

  return(profile_comparison)
}
```

```
# ----------


# define data: AMR profile of 18-SA00377
querydata.file <- "file/path/to/AMR/profile/of/18-SA00377/summary_amrfinder.tsv"

# define accession number for 18-SA00377 in order to exclude it from results
because has been uploaded to ncbi
query_accession <- "SAMEA5882645"

# on 31.10.2022 downloaded  latest AMR data from five different databases ( for
S. enterica, E. coli and Shigella sp., Klebsiella pneumoniae, Enterobacter sp.,
Acinetobacter baumannii)
#from NCBI Pathogen Detection pipeline via their ftp server (e.g.
https://ftp.ncbi.nlm.nih.gov/pathogen/Results/Salmonella/latest_snps/AMR/)

#from here on, only show code for workings using the NCBI database for
Salmonella, but for other four databases, the identical code but with different
variables and respective databases was executed:
amrmetadata.file <-
"file/path/to/downloaded/AMR/data/for/Salmonella/PDG000000002.2498.amr.metadata.
tsv"
amrmetadata <- read.delim(amrmetadata.file, stringsAsFactors = F, quote = "")
nrow(amrmetadata)

# overview of amr data for Salmonella isolates in NCBI Pathogen Detection
pipeline
hist(amrmetadata$number_amr_genes)
max(amrmetadata$number_amr_genes, na.rm = T)
sum(amrmetadata$number_amr_genes > 15, na.rm = T)
mean(amrmetadata$number _amr_genes >= 23, na.rm = T)

# filter Salmonella database for isolates with large number of resistances (i.e.
here 15 ARGs)
amrmetadata_highres <- amrmetadata[amrmetadata$number_amr_genes > 15,]
amrmetadata_highres <- amrmetadata_highres %>% filter(biosample_acc !=
query_accession) # remove self

# define query resprofile of isolate
querydata <- read.delim(querydata.file, stringsAsFactors = F)
query_profile_raw <- querydata$amr_genes[1]
query_profile <- strsplit(query_profile_raw,";")[[1]]
query_profile_count <- length(query_profile)

# query data from ncbi
amrmetadata_self <- amrmetadata %>% filter(biosample_acc == query_accession)
self_genotype_clean <-
strsplit(gsub('\"',"",amrmetadata_self$AMR_genotypes),",")[[1]]
self_genotype_clean <- sapply(self_genotype_clean, function(x)
strsplit(x,"=")[[1]][1])

# compare ARG profile of our isolate to the Salmonella database
profile_comparison <- compare_profiles (amrmetadata_highres =
```

```
amrmetadata_highres, query_profile = self_genotype_clean)

# visualise results of ARG profile comparison
hist(profile_comparison_ncbiversion$matching_genes_relative)
max(profile_comparison_ncbiversion$matching_genes_relative)
sum(profile_comparison_ncbiversion$matching_genes_relative >= 0.8) #determine
how many Salmonella isolates in the database share more than 80% of the same
ARGs as our isolate
neighbors <- which(profile_comparison_ncbiversion$matching_genes_relative >=
0.8)
profile_comparison[neighbors,]

#had to define outdir
outdir = "/file/path/to/output/directory"

#write output tables of the metadata (matrix, geographical origin, serovar) of
the Salmonella isolates that share more than 80% of same ARGs as our isolate
knitr::kable(profile_comparison[neighbors,c("epi_type","geo_loc_name","isolation
_source","serovar")])
write.table(profile_comparison[neighbors,],file.path(outdir,"profile_comparison_
Salmonella.tsv"), sep = "\t", quote = F, row.names = F)
saveRDS(profile_comparison[neighbors,],file.path(outdir,"profile_comparison.rds"
))


#write output tables of the AMR metadata of the Salmonella isolates that share
more than 80% of same ARGs as our isolate
neighbors_biosample <- profile_comparison[neighbors,"biosample_acc"]
amrmetadata_neighbors <- amrmetadata_highres %>% filter(biosample_acc %in%
neighbors_biosample)
write.table(amrmetadata_neighbors,file.path(outdir,"amrmetadata_neighbors_Salmon
ella.tsv"), sep = "\t", quote = F, row.names = F)
saveRDS(amrmetadata_neighbors,file.path(outdir,"amrmetadata_neighbors_Salmonella
.rds"))

# if there are any Salmonella isolates that share more than 80% of same ARGs as
our isolate, downloading their genomes requires their genbank accession numbers:
write(amrmetadata_neighbors$asm_acc,
file.path(outdir,"amrmetadata_neighbors_Salmonella_genbankacc.txt"))
```
