# Supplementary figures and images for: Genetic characterization of a multidrug-resistant Salmonella enterica serovar Agona isolated from a dietary supplement in Germany
Source: Front Microbiol. 2023 Nov 15;14:1284929. doi: 10.3389/fmicb.2023.1284929 (PMC10686068; doi:10.3389/fmicb.2023.1284929)

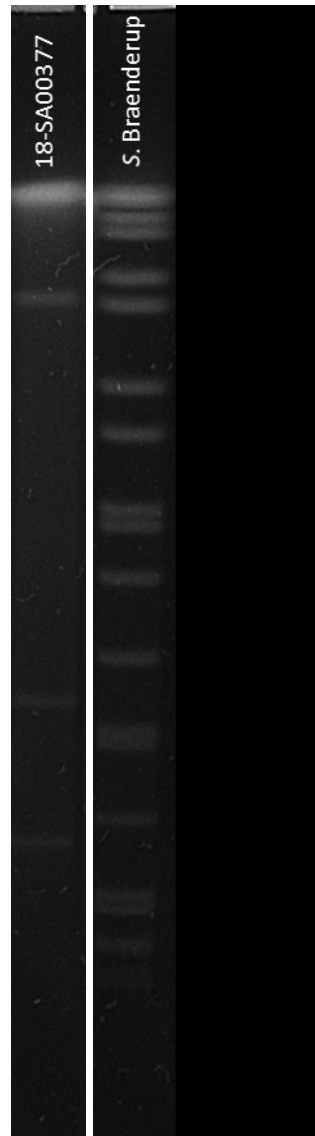

**Supplementary File 1.**S1-PFGE Gel of *S. Agona* (18-SA00377) used with *S. Braenderup* H9812 as size marker.

Supplement: Supplementary file 1 [file Data_Sheet_1.zip › 1284929_Fischer_Data_Sheet_2.PDF]
